# Ovarian reserve alteration in premenopausal women with systemic sclerosis

**DOI:** 10.1007/s00296-024-05724-z

**Published:** 2024-09-25

**Authors:** A. C. Pecher, J. C. Henes, A. Demin, E. M. Staufenberg, M. Henes

**Affiliations:** 1 Centre for Interdisciplinary Clinical Immunology, Rheumatology and Autoinflammatory Diseases, University Hospital Tübingen, Otfried-Mueller-Strasse 10, 72076 Tübingen, Germany; 2 Eberhard-Karls-University, Tuebingen, Germany; 3 Department of Obstetrics and Gynecology, University Hospital Tübingen, Calwerstrasse 7, 72076 Tübingen, Germany; 4 Present Address: University Hospital Tuebingen, Otfried-Mueller-Strasse 10, 72076 Tuebingen, Germany

**Keywords:** Systemic sclerosis, Autoimmune diseases, Fertility, Anti-Muellerian hormone

## Abstract

Anti-Muellerian hormone (AMH) is produced by the granulosa cells of ovarian follicles. It serves as a sensitive laboratory parameter for assessing ovarian reserve. A reduced ovarian reserve has been observed in patients with various autoimmune diseases. To compare serum levels of AMH as a surrogate parameter for ovarian reserve in female patients with systemic sclerosis compared to healthy controls and thereby assess fertility. In this single centre study from the University Hospital Tuebingen, Germany, we used serum samples to determine concentrations of AMH via an electrochemiluminescence immunoassay. We analysed 30 premenopausal female patients with systemic sclerosis and 30 age-matched healthy controls from 18 to 40 years. Patients who had received cyclophosphamide were excluded from this study. AMH levels were significantly reduced in patients with systemic sclerosis (955 ng/l versus 1.940 ng/L, p < 0.01). Interestingly, in contrast to healthy controls, we observed no significant correlation between age and AMH levels in patients. For women diagnosed with systemic sclerosis, especially at a younger age, regular assessment of AMH levels should be considered to improve guidance with regard to optimal pregnancy timepoint, fertility preservation and treatment options.

## Introduction

Anti-Muellerian hormone (AMH) is s a glycoprotein produced by the granulosa cells of ovarian follicles. It serves as a sensitive laboratory parameter for assessing ovarian reserve, which refers to the number and quality of a woman’s remaining eggs. Furthermore, AMH levels correlate with the antral follicle count and are relatively stable throughout the menstrual cycle. Therefore, it is an important factor in estimating individual reproductive potential.

In the context of fertility, AMH levels provide valuable insights into a woman's reproductive lifespan and her response to fertility treatments. Low AMH levels suggest diminished ovarian reserve, often associated with reduced fertility and poorer outcomes in assisted reproductive technologies such as in vitro fertilization (IVF) [1, 2].

In severe cases of systemic sclerosis (SSc) with a poor prognosis, cyclophosphamide (CYC) and high-dose chemotherapy followed by autologous stem cell transplantation (ASCT) are used for treatment. The latest guidelines of the European League Against Rheumatism (EULAR) for SSc have also included ASCT in the recommendations for the treatment of severe lung and skin involvement [3]. Nevertheless, intensive therapies also have a downside, and the gonadal toxicity of CYC, for example, has long been known in patients with haematologic and rheumatic diseases [4, 5]. Furthermore, disease activity and other immunosuppressants might also adversely affect ovarian function. Higher rates of nulliparity and a lower number of children in patients with SSc have been described [6, 7]. Assessing AMH levels in these patients can help healthcare providers tailor fertility preservation strategies and optimize reproductive health management.

As many rheumatic diseases affect women at child bearing age, reproductive health is an important quality of life issue and has been addressed in the past [8–11]. Our group demonstrated a reduced ovarian reserve in patients with systemic lupus erythematosus, rheumatoid arthritis, spondyloarthritis, and behçet's disease [10, 12]. This has also been shown for patients with SSc, especially when they were exposed to CYC in the past [13]. Its impact on reproduction and fertility however has not been elucidated.

Aim of this study was to investigate ovarian reserve by AMH in female SSc patients at a reproductive age who never received CYC and compare them to an age matched healthy control.

## Patients and methods

### Patients and healthy controls

This monocentric study addresses ovarian reserve measured by AMH levels and clinical fertility parameters of female patients with SSc who were treated at the Department of Internal Medicine II, Tuebingen University Hospital, Germany from 2011 to 2023 in collaboration with the Department of Obstetrics and Gynaecology. Thirty-three consecutive patients were screened, and 30 Patients were eligible fulfilling all of the following inclusion and exclusion criteria: fulfilling the ACR-EULAR classification criteria for SSc [14], age between 18 and 40 years, no prior radiation therapy, no prior CYC therapy, no prior chemotherapy due to malignant disease, and normal renal function by creatinine clearance. Medical record data extraction included ongoing immunosuppressants and other disease related medication: prednisone, mycophenolate mofetil (MMF), azathioprine (AZA), methotrexate (MTX), sildenafil, bosentan and for typical anti-nuclear antibodies (ANA): anti-topoisomerase I (Scl-70), anti-centromere (ACA), anti-RNA Polymerase III and anti-PM/Scl. All patients answered a questionnaire on lifestyle and fertility and reproductive data.

Age-matched healthy controls (HC) were recruited in the Department of Obstetrics and Gynaecology.

### Main outcome variable

This study investigates the ovarian reserve from using the estimation of AMH in female patients with SSc at a reproductive age who never received CYC.

### Procedures

Serum for AMH was separated and stored at -20°C until used for assay. AMH was measured in duplicates by electrochemiluminescence immunoassay (ECLIA; Roche Diagnostics, Mannheim, Germany) according to the manufacturer’s protocol. The test measures concentrations between 0.01 and 23 ng/ml. Depending on age, normal values range from 0.05 to 9.49 ng/ml for women. Values < 0.05 ng/ml are considered an indicator for limited ovarian reserve.

### Statistical analysis

Statistical analysis was performed using GraphPad Prism version 10. Variables were compared between patients and controls using the Mann–Whitney test for AMH levels as this value did not show normal distribution in the Shapiro–Wilk test. p < 0.05 was considered statistically significant.

### Ethical statement

The study was approved by the local ethics committee of the Eberhard-Karls-University Tuebingen (IRB 454/2023BO2) and all participants gave their written informed consent.

## Results

Table 1 summarizes the cohort’s characteristics. Median age at time of AMH assessment was 32 years (min–max: 18–40), median disease duration was 28 months (min–max: 1–233), and n = 17 (56%) of the patients showed Scl70 positivity and n = 13 (43%) were diagnosed as dcSSc with a median mRSS of 16 (min–max: 0–40). Regarding the treatment for SSc at the time of AMH measurement: n = 9 (30%) of the patients were taking low-dose prednisone (i.e. ≤ 5mg prednisone per day), n = 8 (27%) MTX, n = 6 (20%) MMF, n = 3 (10%) AZA and n = 5 (17%) bosentan.

**Table 1:** Patients ‘ characteristics

Characteristic	All SSc patients
Fertility parameters	
Age at measurement (years), median (min–max)	32 (18–40)
Number of children, median (min–max)	0 (0–2)
Age at first child (years), median (min–max)	30 (21–35)
Timing of menarche, (years), median (min–max)	13 (9–20)
Regularity in menstruation Regular cycle (no.[%]) Unregular cycle (no.[%]) Amenorrhea (no.[%])	23 (77) 6 (20) 1 (3)
Unfulfilled child wish (no.[%])	7 (23)
Misscarriage (no.[%])	3 (10)
Smoking Ongoing (no.[%]) Never (no.[%])	2 (7) 28 (93)
Disease characteristics	
Disease duration (months), median (min–max)	28 (1–233)
Disease manifestation (no.[%]) dcSSc lcSSc Overlap	13 (50) 15 (43) 2 (6)
Autoantibody (no.[%]) None Scl70 ACA Other	6 (20) 17 (57) 5 (17) 4 (6)
mRSS, median (min–max)	7 (0–40)
Lung involvement (no.[%])	18 (60)
Heart involvement (no.[%])	2 (7)
Treatment at measurement*	
Mycophenolate mofetil	6 (20)
Prednisone	9 (30)
Azathioprine	3 (10)
Methotrexate	8 (27)
Sildenafil	1 (3)
Bosentan	5 (17)

ACA, anti-centromere antibody, dcSSc, diffuse cutaneous systemic sclerosis; lcSSc, limited cutaneous systemic sclerosis; max, maximum; min, minimum; mRSS, modified Rodnan skin score; no., number; Scl70, anti-topoisomerase II; SSc, systemic sclerosis

### Fertility and reproduction

Median age at menarche was 13 years (min–max: 9–20), most (n = 23; 77%) of the patients reported a regular cycle and about one third (n = 10; 33%) of the patients had children (median 1; min–max: 0–2) with the median age of 29.5 years (min–max: 21–35) at the birth of the first child. Three patients (10%) reported of a prior miscarriage and n = 7 (23%) of a child wish but involuntary non-conception.

### AMH levels in patients with systemic sclerosis compared to healthy controls

**Fig. 1:**
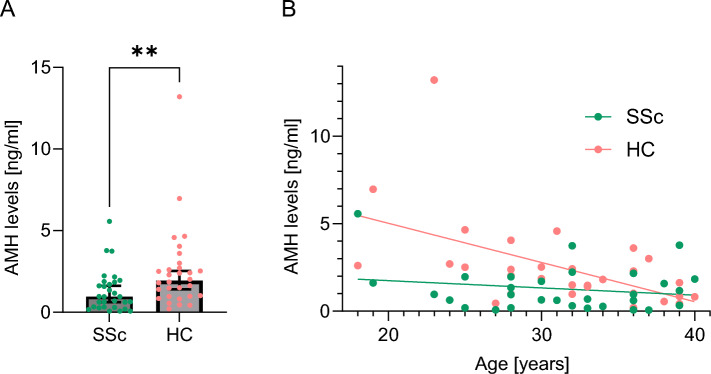
Serum levels of Anti-Muellerian hormone (AMH) in 30 patients with systemic sclerosis (SSc) and 30 age-matched healthy controls (HC). **A** AMH levels were significantly lower in patients with SSc compared to HC (0.955 ng/l versus 1.940 ng/L, p < 0.01). **B** AMH concentration showed an inverse correlation between age at the timepoint of AMH measurement in HC (R^2^ = 0.282, p < 0.01) but not in SSc (R^2^ = 0.038, p = 0.34)

Overall AMH levels were significantly reduced in patients with SSc compared to age-matched HC (median value of 0.955 ng/l [min 0.065 to max 5,57] versus 1.940 ng/l [0.203–13.21], p < 0.01) (Fig. 1A). None of the patients showed AMH levels < 0.05 ng/ml.

### Comparative analysis of AMH levels and clinical parameters

As expected, AMH serum levels inversely correlated with age in HC (R^2^ = 0.282, p < 0.01), this was however not the case for women with SSc (R^2^ = 0.038, p = 0.34).

**Fig. 2:**
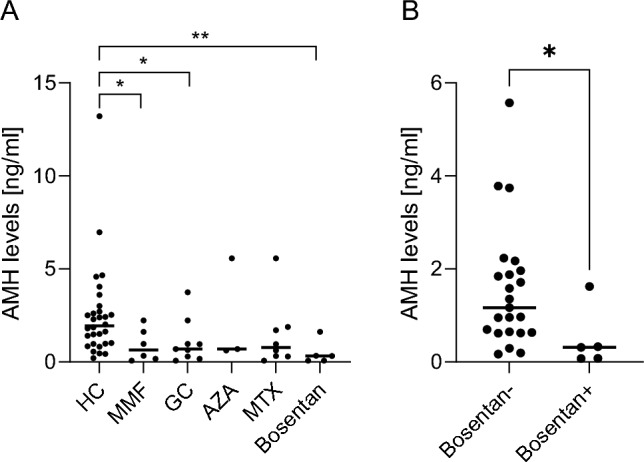
**A/B** Serum levels of Anti-Muellerian hormone (AMH) in patients with systemic sclerosis receiving different therapies (myophenolate mofetil [MMF], glucocorticoids [GC] as high as prednisone ≤ 5mg per day, azathioprine [AZA], and methotrexate [MTX]) compared to healthy controls (HC)) (2**A**). In addition patients with or without bosentan showed a significant difference (2**B**)

To determine, whether immunosuppressants might influence AMH levels, we stratified patients for different medications (patients who were exposed to CYC were excluded in this study): MMF (n = 6), glucocorticoids (GC; n = 9) as high as prednisone ≤ 5mg per day, AZA (n = 3), MTX (n = 8), sildenafil (n = 1) and bosentan (n = 5). Patients who received MMF, GC or bosentan showed significantly lower levels to HC (Fig. 2A). When considering only patients with or without a specific medication, only patients with or without bosentan showed a significant difference (Fig. 2B, non-significant data not shown).

Patients who were not able to have children despite the desire to become pregnant for ≥ 12 months showed comparable levels of AMH (0.96 ng/ml versus 0.95 ng/ml, difference not significant).

## Discussion

Different authors have shown that women with rheumatic diseases often exhibit reduced AMH levels compared to healthy controls [10, 12, 15, 16], but few studies have been executed to assess fertility in patients with SSc. Our study demonstrates that also in premenopausal patients with SSc the serum AMH level is diminished compared to healthy controls. Lower AMH levels suggest a reduced ovarian reserve, which can affect fertility. We therefore conclude, that women with SSc may face challenges in conceiving and may require fertility preservation strategies. Similar results were obtained by Goloeva et al. [13], however they also included patients who had received CYC. Since CYC is known to impair fertility, the authors were unable to distinguish between the potential association of CYC or disease-related factors and reduced AMH levels. Our data therefore confirm a diminished ovarian reserve in patients with SSc even without the known negative impact of prior therapy with CYC.

Several factors might contribute to AMH reduction in patients with SSc: Chronic inflammation associated with SSc might negatively impact ovarian function and lead to lower AMH levels. Furthermore, fibrosis and vascular damage, the pathologic hallmarks of SSc, might also affect the ovarian tissue which could lead to impaired ovarian function and reduced AMH levels.

For women diagnosed with SSc, especially at a younger age, regular assessment of AMH levels and fertility preservation techniques such as egg or embryo freezing should be considered. Hormonal replacement therapy may be beneficial for managing symptoms of primary ovarian insufficiency and improving quality of life, though it must be carefully weighed against potential risks, especially in autoimmune conditions [17, 18].

Increased risk of miscarriage has been reported in some, but not all analyses of patients with SSc [6, 19, 20]. In our analysis three patients (10%) reported of a prior miscarriage which is comparable to the general population [21]. However, we observed a high percentage of patients with child wish but involuntary non-conception (23%, n = 7) whereas the number of the general population is usually estimated to be low at about 5% [22, 23]. We did not observe a significant difference in between women with sustained or fulfilled child wish, suggesting that also other factors might influence the high number.

Our study has limitations, as this is a small single centre cohort and therefore generalizability might be limited. The small sample sizes can lead to a lack of statistical power, therefore interpretation of AMH levels in the different medication groups is only possible to a limited extent. More research is needed to confirm these results and understand the underlying mechanisms leading to reduced AMH levels in SSc. Therefore, also longitudinal follow-ups, investigating the role of immunosuppressive treatments and their impact on ovarian reserve are essential.

Reduced AMH levels in patients with systemic sclerosis highlight the intersection between autoimmune disease and reproductive health. Recognizing and addressing this issue is crucial for improving the overall well-being and quality of life of women with SSc. A proactive approach, including early diagnosis of the fertility status, e.g. by estimation by AMH levels will help us when counselling SSc patients especially with regard to (early) fertility preservation, such us social freezing, optimal timepoint for pregnancy and also with regard to therapeutic strategies by using more targeted therapies instead of CYC. This will mitigate the reproductive challenges faced by these patients. Further research will continue to elucidate the complex relationship between SSc and ovarian function, paving the way for better management strategies in the future.
